# A Multifaceted Nursing Practice Bundle for the Management of Changed Behaviour in Hospitalised Adults With Cognitive Impairment: A Pre‐/Poststudy

**DOI:** 10.1111/ajag.70225

**Published:** 2026-08-27

**Authors:** Erin Rachel Davis, Christina Aggar, Michelle Bissett

**Affiliations:** ^1^ Southern Cross University Bilinga Queensland Australia; ^2^ Northern NSW Local Health District Northern NSW Australia

**Keywords:** cognitive dysfunction, delirium, dementia, hospital nursing staff

## Abstract

**Objective:**

The aim of this study was to evaluate changes in nursing practices following implementation of a multifaceted nursing practice bundle for the management of changed behaviour in hospitalised adults with cognitive impairment.

**Methods:**

A single‐group pre–post interventional study evaluated changes in nurses' reactions to changed behaviour and the use of alternatives to physical restraint following implementation of a multifaceted nursing practice bundle (implementation framework, nurse education and nonpharmacological resources).

**Results:**

Management of changed behaviour improved, with nurses more commonly reacting with care and less likely to react by ignoring when changed behaviour occurred. Nurses were also more likely to utilise professional knowledge and problem‐solving abilities, and less likely to use medications as alternatives to physical restraints.

**Conclusions:**

The implementation of a nursing practice bundle was associated with improved management of changed behaviour in hospitalised adults with cognitive impairment, likely due to its multifaceted approach. Improvements in nursing practice indicate enhanced attitudes towards adults with cognitive impairment and increased confidence in implementing nonpharmacological interventions.

## Impact Statements

This study contributes to the growing body of research evaluating changes in nursing practice to support adults with cognitive impairment in hospital following implementation of a multifaceted bundle. Findings suggest that organisationally driven multifaceted bundles may support improvements in nursing practice.

## 1 Introduction

Cognitive impairment is a deficit in thinking, learning and remembering that may impact a person's judgement, decision‐making and concentration [1, 2]. Cognitive impairment in adults is commonly associated with dementia and delirium, with rates increasing alongside an ageing population [3]. Consistent with global trends [2], Australia has seen a 22% increase in hospital admissions of adults with dementia in the past decade [4]. Delirium rates have also risen, with a known association with dementia [1]. This trajectory is likely to continue, as the number of older adults is predicted to increase by a factor of three by 2050 [2].

Adults with cognitive impairment are more likely to experience longer hospital stays and hospital‐acquired complications such as deconditioning, falls, infections, dehydration, reduction in nutritional status and pain [5, 6], contributing to higher rates of mortality and institutionalisation [1]. Hospital‐acquired complications are in part due to the complex nature of providing care to adults with cognitive impairment, with cognitive and functional decline linked to the occurrence of changed behaviour, which may impact nurses and established clinical routines [1]. This paper focusses on improving nursing practices through increased utilisation of person‐centred nonpharmacological interventions to better support adults with cognitive impairment in hospital.

## 2 Background

Hospitalisation of adults with cognitive impairment is linked to an exacerbation of changed behaviour [5], such as aggression, apathy, psychomotor agitation and declining care [7, 8]. Described as an active attempt to express or address an unmet need [9, 10], these behaviours indicate underlying distress, anxiety and agitation. Common causes of unmet needs in adults with cognitive impairment have been identified as a biologically lowered stress threshold, exacerbated by the suboptimal hospital environment and rigid routine, as well as communication deficits [7, 10]. Chemical and physical restraints are often used when changed behaviours occur in hospital, contributing to increased morbidity and mortality [11, 12]. International guidelines recommend nonpharmacological interventions in preference to chemical and physical restraints [13, 14]. Aligned with these guidelines, nursing practices that are person‐centred and consider the individual's needs, preferences and lifestyle are endorsed [15].

Nonpharmacological interventions underpinned by person‐centred care can reduce the unmet needs of adults with cognitive impairment and improve quality of life [7]. Nonpharmacological interventions have proven effective in a variety of care settings [7] but are underutilised in hospital [16]. Nurses are well‐placed to deliver nonpharmacological interventions, but barriers to implementation include prioritising acute care needs, time and resource constraints, understaffing, under‐recognition of cognitive impairment and limited continuity of guiding frameworks [8, 17, 18]. Additionally, nurses commonly report negative attitudes towards adults with cognitive impairment and doubts regarding the efficacy of nonpharmacological interventions to manage changed behaviour [19]. Education has been demonstrated to effectively overcome negative attitudes and increase the use of nonpharmacological interventions [20, 21]. In addition to education, providing resources and a framework to guide person‐centred care, inclusive of nonpharmacological interventions, may further address reported barriers [5, 16, 22]. Previous Australian initiatives have sought to improve hospital care for people with cognitive impairment, including the Dementia Care in Hospitals Program [23] and other multicomponent interventions [11, 16, 24, 25, 26]. However, the evidence base regarding multifaceted nursing practice bundles targeting changed behaviours in hospitalised adults with cognitive impairment remains relatively small. This study contributes to this emerging body of evidence and supports ongoing program design and implementation.

## 3 The Study

### 3.1 A Multifaceted Nursing Practice Bundle

A multifaceted nursing practice bundle to improve the management of changed behaviour in adults with cognitive impairment was co‐designed by researchers, nurses, patients and family carers. Theoretically underpinned by the Unmet Needs Model [9, 10], the bundle consisted of three complementary elements: (i) a health district‐initiated implementation framework, (ii) a nurse education session and (iii) nonpharmacological resources. The framework categorised changed behaviour (mild confusion, mild agitation, moderate agitation, severe agitation), with corresponding recommendations for nonpharmacological interventions in line with the health district's policies and guidelines (available from the corresponding author on request). The framework was introduced into the education sessions using realistic hospital‐focussed case studies. Education sessions emphasised the underlying causes of changed behaviour and the importance of person‐centred care, enabling nurses to recognise and respond to it. Nonpharmacological resources (fidget mats and boards) were provided to each participating ward to support the application of interventions recommended in the framework and reinforced through education. Other nonpharmacological interventions suggested in the framework, such as listening to music, were facilitated by preexisting ward resources. Together, these components were intended to guide assessment, decision‐making and implementation of person‐centred nonpharmacological interventions to improve management of changed behaviour in adults with cognitive impairment.

### 3.2 Objective

The aim of this study was to evaluate changes in nursing practices following implementation of a multifaceted nursing practice bundle for the management of changed behaviour in hospitalised adults with cognitive impairment.

## 4 Methods

### 4.1 Ethical Considerations

Ethics approval was granted for this study by the Northern NSW Local Health District Research Office (QA455; 2023/124).

### 4.2 Design

A single‐group pre‐/postinterventional study using surveys was designed to address the research question: *Does implementation of a multifaceted nursing practice bundle change how nurses manage changed behaviour in hospitalised adults with cognitive impairment?* An amended version of the psychometrically tested tool developed by Hynninen et al. [27] was used. The tool was originally developed to measure nurses' self‐reported practices when caring for people with dementia in an acute hospital surgical ward and has subsequently been used to explore nurses' self‐reported practices for adults with cognitive impairment in hospital [27, 28] and in aged care [29]. The tool does not appear to have been used in any previous pre‐/poststudies.

The Hynninen et al. [27] tool consisted of three subscales to measure nurses' self‐reported practices for adults with dementia on a surgical ward: c*ounselling people with dementia in the surgical ward, reaction when a patient with dementia displays changed behaviour and use of alternative approaches instead of physical restraints*. This study adopted two subscales relevant to its aim, which supported the pre‐/postexploration of nursing practices. Similarly, amended versions have been previously used by Keuning‐Plantinga et al. [28] and Piirainen et al. [29] The first subscale *reaction when a patient with cognitive impairment displays changed behaviour* consists of four factors: (i) react with care (four items), (ii) react by ignoring (two items), (iii) react with power (three items) and (iv) react casually (two items). The second subscale *use of alternate approaches instead of physical restraints* consists of three factors: (i) use of professional knowledge (five items), (ii) use of medication (two items) and (iii) use of problem‐solving (two items). A Likert scale enabled participants to self‐report frequency of use for each practice (1 = never to 5 = very often). Demographic data were also collected, including participant employment position and type, years of experience and age. The Strengthening the Reporting of Observational Studies in Epidemiology (STROBE) statement has been applied to this study (File S1) [30].

### 4.3 Participants

The intervention was implemented on 13 acute and subacute wards across nine Australian regional and rural hospitals, selected for their high volume of patients with cognitive impairment. Participants were assistants in nursing, enrolled nurses and registered nurses (collectively referred to as nurses throughout this paper). The sample size was opportunistic, aiming to include as many nurses as possible within the available time.

### 4.4 Intervention Implementation

The multifaceted nursing practice bundle was co‐designed by the local health district prior to this study; this research focussed solely on its implementation and evaluation. From August to November 2023, a total of 50 education sessions were delivered to nurses on shift during designated in‐service education time. The education package involved a 30‐min face‐to‐face session delivered by specialised senior nurses, using a standardised PowerPoint presentation. Nurses were instructed to use the framework to guide assessment of changed behaviour and to select appropriate nonpharmacological interventions based on individual patient needs. The framework and resources were disseminated after education was completed across all sites, ensuring that all participants received foundational education before accessing the resources. Nonpharmacological resources were funded by the local health district and assembled by local community groups.

### 4.5 Data Collection

Before commencement of the education sessions, nurses were provided with a copy of the Participant Information Statement and invited to anonymously complete a paper‐based or online survey hosted by Qualtrics XM via a quick‐response (QR) code. Three to 6 months later, nurses were invited to complete an anonymous online follow‐up survey. A reminder message was sent at 2 weeks to prompt participant response to the survey. Pre‐ and postintervention surveys were matched using a unique code generated by the participant (the last four digits of the participant's phone number and the first initial of their mother's name).

### 4.6 Data Analysis

Data were analysed using IBM SPSS Statistics (version 29). Matched surveys were included if both pre‐ and postsurveys were over 80% complete. Before analysis, reverse scoring was applied to items representing less desirable practices to ensure consistency within subscales, such that higher scores indicated more desirable nursing practices.

Descriptive statistics were used to report participant demographics. Wilcoxon matched‐pair signed‐rank (two samples) was used to analyse changes in the surveys pre and post, with an alpha of 0.05 considered significant. Changes in individual items were calculated first (File S2) and then aggregated to calculate factor level changes (Table 3), the results of which were prioritised in this study. As the Wilcoxon test automatically removes ties, the mean and median were calculated to show the overall pattern of change for each factor as assigned by Hynninen et al. [27] Cronbach's alpha was used to assess the internal reliability of the tool following data collection.

## 5 Results

### 5.1 Participant Demographics

Presurvey responses were obtained from nurses across 13 wards at nine sites. Postsurvey responses were obtained from nurses across 12 wards at eight sites, indicating representation across most participating wards and sites. In total, 235 nurses completed the presurvey, and 106 nurses completed the postsurvey. Of these, 62 matched surveys were eligible for analysis (Table 1). Surveys could not be matched where unique codes were incomplete or missing; in addition, high staff turnover across sites limited the number of participants available to complete both pre‐ and postintervention surveys.

**TABLE 1:** Distribution of survey respondents across participating sites.

	Wards	Pre (*n* = 235)	Post (*n* = 106)	Matched (*n* = 62)
Site 1	1	23	1	1
Site 2	1	22	8	4
Site 3	1	21	4	4
Site 4	3	37	3	3
Site 5	1	19	11	8
Site 6	1	12	10	5
Site 7	3	78	61	31
Site 8	1	2	0	0
Site 9	1	21	8	6

Participant demographics are presented in Table 2. Most nurses were registered (63%), aged between 35 and 44 years (31%), had 2–6 years' experience (31%) and worked part‐time (55%).

**TABLE 2:** Participant demographics (*n* = 62).

Position	*n*	%
Assistant in Nursing	1	2
Endorsed Enrolled Nurse	12	19
Registered Nurse	39	63
Clinical Nurse Specialist	3	5
Clinical Nurse Educator	7	11
**Age in years**		
< 24	6	10
25–34	14	23
35–44	19	31
45–54	13	21
> 55	10	16
**Work experience (in healthcare) in years**		
< 1	6	10
2–6	19	31
7–15	17	27
16–30	14	23
> 31	6	10
**Type of employment**		
Casual	5	8
Contract	1	2
Part time	34	55
Full time	22	36
Casual	5	8

### 5.2 Changes in Nursing Practice for the Management of Changed Behaviour Following Implementation of a Multifaceted Nursing Practice Bundle

In Subscale 1, *reaction when an adult with cognitive impairment displays changed behaviour*, all factors improved significantly, with significant increases in reacting with care (*p* ≤ 0.001) and *ignoring* (*p* = 0.005). In Subscale 2, *use of alternate approaches instead of physical restraint*, all factors significantly improved (Table 3). Statistically significant findings were associated with small to moderate effect sizes (*r* = 0.19–0.33). Factor item results pre and post are presented in File S2.

**TABLE 3:** Factor changes pre–post.

Subscale one	Median		Mean		Test statistic, *p* score	Effect size
Reaction when an adult with cognitive impairment displays changed behaviour	Pre	Post	Pre	Post		
Factor 1—React with care (*n* = 59)	2.55	2.72	2.50	2.70	*T* = 1237, ***p* ≤ 0.001**	0.33
Factor 2—React by ignoring^a^ (*n* = 61)	3.46	3.80	3.39	3.55	*T* = 486.5, ***p* = 0.005**	0.26
Factor 3—React with power^a^ (*n* = 59)	2.34	2.39	2.37	2.42	*T* = 626.5, *p* = 0.06	0.17
Factor 4—React casually (*n* = 59)	1.79	1.79	1.73	1.79	*T* = 648.5, *p* = 0.24	0.11

Subscale two	Median		Mean		Test statistic, *p* score	Effect size
Use of alternate approaches instead of physical restraints	Pre	Post	Pre	Post		
Factor 1—Use of professional knowledge (*n* = 59)	2.78	2.93	2.81	2.96	*T* = 999.5, ***p* = 0.005**	0.26
Factor 2—Use of medications^a^ (*n* = 60)	2.43	2.66	2.51	2.60	*T* = 536.0, ***p* = 0.04**	0.19
Factor 3—Use of problem‐solving (*n* = 62)	2.75	3.14	2.93	3.11	*T* = 681.5, ***p* = 0.004**	0.26

*Note:* Bold results represent those with significant *p* value.

^a^ Reverse scoring was applied.

### 5.3 Validity and Reliability/Rigour

Cronbach's alpha was used to measure the internal reliability of both subscales pre and post. Internal reliability for predata was considered acceptable for subscale 1 (*α* = 0.63) and good for subscale 2 (*α* = 0.77). Postdata reliability was significantly lower for Subscale 1 (*α* = 0.49) and Subscale 2 (*α* = 0.70). This change in internal reliability suggests that responses to certain items may have changed following implementation of the multifaceted nursing practice bundle, but not consistently across all items within the factor.

## 6 Discussion

The implementation of a multifaceted nursing practice bundle was associated with improved management of changed behaviour in hospitalised adults with cognitive impairment. These changes suggest a shift towards practice that is more consistent with current Australian nursing standards and international recommendations [13, 14, 31, 32]. Specifically, when changed behaviours occurred, nurses indicated that they more often *react with care* and are less likely to *react by ignoring*. Nurses also reported that, as alternatives to physical restraints, they were more likely to use *professional knowledge* and *problem‐solving abilities* and less likely to endorse *medication use*. These changes may reflect the multifaceted approach of the bundle and are likely underpinned by enhanced nursing attitudes towards adults with cognitive impairment and increased confidence in implementing nonpharmacological interventions.

The multifaceted nursing practice bundle in this study included a health district‐initiated implementation framework, nurse education and nonpharmacological resources. The findings of our study contribute to the body of literature supporting multifaceted approaches to enhance nursing practices [11, 16, 33]. Programs that incorporate multifaceted strategies to target multiple barriers are known to be more effective in addressing complex healthcare issues than single strategy methods [34]. This is exemplified by research in quality improvement [35], pressure injury prevention [36] and fall prevention [37]. The positive results observed in our study are consistent with previous research, which explored the feasibility [38] and broader impact [39] of multifaceted nonpharmacological programs to support adults with cognitive impairment in hospital. Discussion of the underlying factors influencing nursing practice may offer a more nuanced understanding of changes following implementation of the bundle.

A critical relationship exists between nursing attitudes, confidence and practice [8, 21]. Negative nursing attitudes towards adults with cognitive impairment are common, linked to reduced confidence in providing care [19] and increased use of restraints [33]. Conversely, enhancing nurses' attitudes and confidence to support adults with cognitive impairment and manage when changed behaviour occurs has been shown to increase person‐centred care [5, 8, 21] and effective use of nonpharmacological interventions [12]. Organisational supports, including a framework [16], education [19, 21] and nonpharmacological resources [5, 22], have previously been identified as key strategies in improving nursing attitudes and confidence. Consequently, the improvements to nursing practice observed in our study indicate enhanced underlying attitudes and confidence when caring for adults with cognitive impairment. Future research examining the impact of these improvements on patient‐level outcomes is needed to strengthen the link between nursing practice and care quality and to better understand the contribution of multifaceted nursing practice bundles to patient outcomes.

### 6.1 Strengths and Limitations

A strength of this study is its contribution to the limited quantitative evidence on multifaceted nursing practice bundles for managing changed behaviour in hospital settings, demonstrating improvements in nurses' real‐world practice. Furthermore, social desirability bias from nursing participants was likely reduced through maintaining anonymity in the surveys.

Several limitations should be considered when interpreting the findings. Generalisability may be limited by sampling from a single regional health district and a small sample size, with survey attrition potentially reducing estimate precision. Self‐selection may also be present among nurses who participated in the postsurvey. The study did not include direct measures of utilisation of the bundle components, limiting the ability to determine the extent of uptake in practice. The magnitude of change observed was small to moderate, and, given the relatively small, matched sample, the findings should be interpreted with caution. Finally, the internal reliability of subscale one was low, potentially affecting the validity of the results. The low internal reliability of the Hynninen et al. [27] tool was also reported by Keuning‐Plantinga et al. [28], indicating that further testing is required.

### 6.2 Implications for Policy and Practice

Nursing practice aligned more closely with international recommendations for person‐centred nonpharmacological interventions following implementation of a multifaceted nursing practice bundle. These findings support consideration of multifaceted programs within policy and practice initiatives aimed at improving care for adults with cognitive impairment.

## 7 Conclusions

After implementing a multifaceted nursing practice bundle, nurses reported improved management of changed behaviour in hospitalised adults with cognitive impairment. Incorporating an implementation framework, nurse education and nonpharmacological resources likely contributed to these positive results. Additionally, the results of this study indicate that a multifaceted nursing practice bundle may enhance attitudes towards adults with cognitive impairment and confidence in implementing nonpharmacological interventions.

## Funding

The authors have nothing to report.

## Ethics Statement

Ethics approval was granted for this study by the Northern NSW Local Health District Research Office (QA455; 2023/124).

## Conflicts of Interest

The authors declare no conflicts of interest.

## Supporting information


**File S1:** STROBE Statement—checklist of items that should be included in reports of observational studies.

**File S2:** Item changes pre–post.

## Data Availability

The data that support the findings of this study are available on request from the corresponding author. The data are not publicly available due to privacy or ethical restrictions.
