# Supplementary material for: A Multifaceted Nursing Practice Bundle for the Management of Changed Behaviour in Hospitalised Adults With Cognitive Impairment: A Pre‐/Poststudy
Source: Australas J Ageing. 2026 Aug 27;45(3):e70225. doi: 10.1111/ajag.70225 (PMC13522401; doi:10.1111/ajag.70225)
Supplement: Supplementary file 1 [file AJAG-45-0-s001.docx]

**Supplementary File 2:** Item changes pre-post

| **Subscale 1** | | **Median** | | **Mean** | | **Test Statistic, *p* score** |
| --- | --- | --- | --- | --- | --- | --- |
| **Reaction when an adult with cognitive impairment displays changed behaviour** | | **Pre** | **Post** | **Pre** | **Post** |  |
| **Factor 1 – React with care** | | | | | | |
| 1 | I ask them what is wrong (n=60) | 4.00 | 4.00 | 4.05 | 4.20 | T = 320.5, *p* = 0.26 |
| 2 | I check the patient’s record for background and possible instructions (n=62) | 4.00 | 4.00 | 4.16 | 4.32 | T = 247.5, *p* = 0.13 |
| 3 | I organize activities for the patient (n=62) | 4.00 | 4.00 | 3.50 | 4.08 | T = 537.5, *p* = <.001 |
| 4 | I’m present for the patient (n=61) | 4.00 | 4.00 | 4.00 | 4.24 | T = 351.0, *p* = 0.03 |
| **Factor 2 – React by ignoring** | | | | | | |
| 5 | I do nothing* (n=61) | 5.00 | 5.00 | 4.36 | 4.73 | T = 250.0, *p* = 0.002 |
| 6 | I pretend not to hear, see or notice anything* (n=62) | 5.00 | 5.00 | 4.36 | 4.73 | T = 165.5, *p* = 0.36 |
| **Factor 3 – React with power** | | | | | | |
| 7 | I take them to their own room* (n=62) | 2.00 | 2.50 | 2.47 | 2.42 | T = 132.0 *p* = 0.58 |
| 8 | I use physical force to calm down the situation* (n=62) | 5.00 | 5.00 | 4.68 | 4.79 | T = 51.0 *p* = 0.09 |
| 15 | I give orders to the patient* (n=62) | 4.00 | 4.00 | 4.03 | 4.25 | T = 232.0, *p* = 0.04 |
| **Factor 4 – React casually** | | | | | | |
| 10 | I deal with humour (n=60) | 3.00 | 3.00 | 3.02 | 2.98 | T = 257.0, *p* = 0.89 |
| 11 | I tolerate the behaviour (n=62) | 3.00 | 3.00 | 2.84 | 3.02 | T = 285.5 *p* = 0.12 |
| **Subscale 2** | | **Median** | | **Mean** | | **Test Statistic, P score** |
| **Use of alternate approaches instead of physical restraints** | | **Pre** | **Post** | **Pre** | **Post** |  |
| **Factor 1 – Use of Professional Knowledge** | | | | | | |
| 12 | I try to make the physical care environment safer (n=61) | 4.00 | 5.00 | 4.44 | 4.61 | T = 160, *p* = 0.02 |
| 13 | I am present for the patient (n=60) | 4.00 | 4.00 | 4.12 | 4.35 | T = 244.5, *p* = 0.02 |
| 14 | I can handle the patient’s challenging behaviour (n=62) | 4.00 | 4.00 | 3.74 | 3.90 | T = 267.0, *p* = 0.11 |
| 15 | I negotiate with my colleagues about the correct response (n=62) | 4.00 | 4.00 | 4.11 | 4.31 | T = 315.0, *p* = 0.07 |
| 16 | I negotiate with the patient’s family about the correct response (n=62) | 4.00 | 4.00 | 3.92 | 4.13 | T = 294.5, *p* = 0.07 |
| **Factor 2 – Use of Medications** | | | | | | |
| 17 | I give sedative medication to the patient* (n=61) | 3.00 | 3.00 | 3.20 | 3.44 | T = 370.0, *p* = 0.03 |
| 18 | I give painkillers to the patient (n=61) | 3.00 | 3.00 | 3.30 | 3.26 | T = 146.0, *p* = 0.90 |
| **Factor 3 – Use of problem solving** | |  |  |  |  |  |
| 19 | I try to draw the patient’s attention elsewhere (n=62) | 4.00 | 4.00 | 3.90 | 4.18 | T = 348.0, *p* = 0.01 |
| 26 | I try to organise expert consultation for the patient (n=62) | 4.00 | 4.00 | 3.55 | 3.74 | T = 304.5*, p* = 0.04 |
| **Reverse scoring was applied* | | | | | | |
